# Ectopic biosynthesis of catechin of tea plant can be completed by co-expression of the three *CsANS*, *CsLAR*, and *CsANR* genes

**DOI:** 10.1093/hr/uhae304

**Published:** 2024-10-30

**Authors:** Ni Yang, Jing-Wen Li, Yuan-Jie Deng, Rui-Min Teng, Wei Luo, Gui-Nan Li, Zhi-Hang Hu, Hui Liu, Ai-Sheng Xiong, Jian Zhang, Quan-Hong Yao, Jing Zhuang

**Affiliations:** Tea Science Research Institute, Ministry of Agriculture and Rural Affairs Key Laboratory of Biology and Germplasm Enhancement of Horticultural Crops in East China, College of Horticulture, Nanjing Agricultural University, 1 Weigang, Nanjing 210095, China; Tea Science Research Institute, Ministry of Agriculture and Rural Affairs Key Laboratory of Biology and Germplasm Enhancement of Horticultural Crops in East China, College of Horticulture, Nanjing Agricultural University, 1 Weigang, Nanjing 210095, China; State Key Laboratory of Crop Genetics & Germplasm Enhancement and Utilization, College of Horticulture, Nanjing Agricultural University, 1 Weigang, Nanjing 210095, China; Tea Science Research Institute, Ministry of Agriculture and Rural Affairs Key Laboratory of Biology and Germplasm Enhancement of Horticultural Crops in East China, College of Horticulture, Nanjing Agricultural University, 1 Weigang, Nanjing 210095, China; Tea Science Research Institute, Ministry of Agriculture and Rural Affairs Key Laboratory of Biology and Germplasm Enhancement of Horticultural Crops in East China, College of Horticulture, Nanjing Agricultural University, 1 Weigang, Nanjing 210095, China; Tea Science Research Institute, Ministry of Agriculture and Rural Affairs Key Laboratory of Biology and Germplasm Enhancement of Horticultural Crops in East China, College of Horticulture, Nanjing Agricultural University, 1 Weigang, Nanjing 210095, China; Tea Science Research Institute, Ministry of Agriculture and Rural Affairs Key Laboratory of Biology and Germplasm Enhancement of Horticultural Crops in East China, College of Horticulture, Nanjing Agricultural University, 1 Weigang, Nanjing 210095, China; State Key Laboratory of Crop Genetics & Germplasm Enhancement and Utilization, College of Horticulture, Nanjing Agricultural University, 1 Weigang, Nanjing 210095, China; State Key Laboratory of Crop Genetics & Germplasm Enhancement and Utilization, College of Horticulture, Nanjing Agricultural University, 1 Weigang, Nanjing 210095, China; Faculty of Agronomy, Jilin Agricultural University, 288 Newtown Street, Changchun, China; Department of Biology, University of British Columbia, 3333 University Way, Okanagan, Kelowna, BC, V1V 1V7, Canada; Biotechnology Research Institute, Shanghai Academy of Agricultural Science, 2901 Beidi Road, Shanghai 201106, China; Tea Science Research Institute, Ministry of Agriculture and Rural Affairs Key Laboratory of Biology and Germplasm Enhancement of Horticultural Crops in East China, College of Horticulture, Nanjing Agricultural University, 1 Weigang, Nanjing 210095, China

##  

Dear Editor,

Tea plant [*Camellia sinensis* nL.) O. Kuntze] is an important woody economic crop. Tea is a widely popular nonalcoholic beverage, with ~2 billion cups consumed worldwide daily. It is renowned for its unique flavor and numerous health benefits [1]. Tea leaves are rich in characteristic metabolites that benefit human health, including tea polyphenols, theanine, and caffeine. Catechins (flavan-3-ols) constitute ~70% of tea polyphenols and are the main bioactive compounds in tea. It is crucial to the sensory quality of tea and has many pharmacological effects including antidiabetes and cancer prevention [2]. Catechins are mainly divided into *cis*-catechins [epicatechin (EC), epigallocatechin (EGC), epicatechin gallate (ECG), epigallocatechin gallate (EGCG)] and trans-catechins [catechin (C), gallocatechin (GC), catechin gallate (CG), gallocatechin gallate (GCG)].

The biosynthesis pathway of catechins (Fig. 1d) has become better understood. Multiple catechin biosynthesis and regulation genes have been cloned and studied [3]. The biosynthesis of catechins begins with phenylalanine and is catalyzed by over a dozen key enzymes [2]. Among them, anthocyanidin synthase (ANS), anthocyanidin reductase (ANR), and leucoanthocyanidin reductase (LAR) are downstream genes in the biosynthesis pathway of catechins. ANR catalyzes anthocyanins to produce epicatechins (EC, EGC), and LAR converts the leucoanthocyanidins to corresponding catechins (C, GC). They may play a significant role in determining the composition of catechins [3–5]. A number of successful experiments have been conducted in plant multigene genetic engineering studies. The contents of riboflavin and vitamins B1 in rice endosperm were increased by metabolic engineering [6, 7]. Also, rice endosperm synthesizes betalains through metabolic engineering [8]. The main objective of this study is to fortify catechin content through genetic engineering. We selected three key genes involved in the biosynthesis pathway of catechins, *CsANS*, *CsLAR*, and *CsANR*, as the metabolic genes for catechin biofortification.

**Figure 1:**
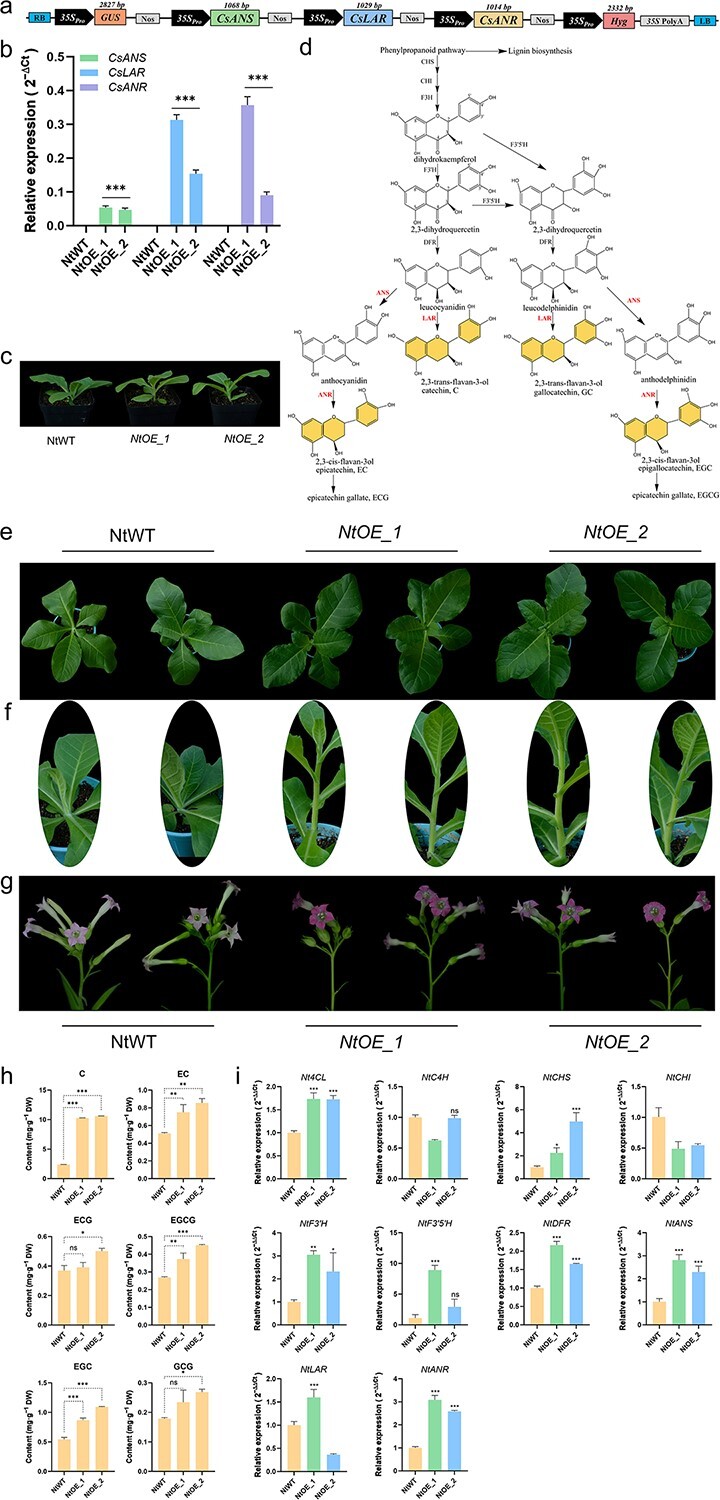
. Co-expression of *CsANS*, *CsLAR*, and *CsANR* in tobacco. (a) Schematic representation of recombinant vectors used in tobacco transformation. (b) Transcriptional expression level of *CsANS*, *CsLAR*, and *CsANR* in wild-type and transgenic tobacco lines by qRT-PCR. Values are mean ± SD of three replicates. (c) Growth phenotypes of the wild-type and transgenic tobacco 20 days after transplanting. (d) Possible biosynthetic pathways of catechins. Genes used for catechin engineering in tobacco are shown in red. (e) Growth phenotypes of 4-week-old wild-type and transgenic tobacco. (f) Stem phenotypes corresponding to the 4-week-old wild-type and transgenic tobacco in (e). (g) Flowers phenotypes of wild-type and transgenic tobacco. (h) Catechins content of wild-type and transgenic tobacco. Catechin (C); epicatechin (EC); epicatechin gallate (ECG); epigallocatechin gallate (EGCG); epigallocatechin (EGC); gallocatechin gallate (GCG). (i) Expression profiles of catechin biosynthesis pathway genes in wild-type and transgenic tobacco. The asterisks (*) indicate the significance level between transgenic tobacco and wild-type tobacco (^*^P < 0.05, ^**^P < 0.01, ^***^P < 0.001).

Firstly, we performed the codons optimization of three genes (*CsANS*, *CsLAR*, and *CsANR*) and carried out the chemical synthesis. Each gene expression unit is connected between the *35S* promoter and the nopaline synthase terminator by a polyacrylamide gel electrophoresis (PAGE)-mediated overlapping extension polymerase chain reaction (PCR) method. The three genes (*CsANS*, *CsLAR*, and *CsANR*) were arranged in series for co-expression, employing the same-end enzyme technique. Subsequently, we transformed the recombinant vector pYC1301 (Fig. 1a) into tobacco. Ultimately, we obtained two transgenic tobacco lines. The growth period phenotype is shown (Fig. 1e), the transgenic tobacco growing strongly overall. The stems of the transgenic tobacco emerge preferentially 20 days after transplanting (Fig. 1c). The growth of the stems in 4-week-old transgenic tobacco is more visually evident (Fig. 1f). More intriguingly, the flowers of the transgenic tobacco exhibit a deeper pink color (Fig. 1g).

We extracted RNA from wild-type and transgenic tobacco to analyze the transcriptional activity of three genes (*CsANS*, *CsLAR*, and *CsANR*) by using quantitative reverse transcription polymerase chain reaction (qRT-PCR) (Fig. 1b). The results showed that the expression of three genes was not detected in wild-type tobacco. In transgenic lines 1 and 2, the inserted three genes (*CsANS*, *CsLAR*, and *CsANR*) were transcriptionally active.

Subsequently, we measured the catechin content of the wild-type and two transgenic lines by ultra-performance liquid chromatography. Several catechin monomers (C, GCG, EC, ECG, EGC, and EGCG) were detected in transgenic tobacco (Fig. 1h). Among them, the content of catechin (C) was the highest. The average catechin (C) content in transgenic tobacco (10.44 mg/g dry weight (DW)) was 4.4-fold higher than the catechin content of wild-type tobacco (2.38 mg/g DW). The EC, EGCG, and EGC content were significantly higher than the wild type, with average increases of 57.8, 51.9, and 82.4%, respectively. Pang’s [3] research indicated that *CsANR* can convert anthocyanins into a mixture of epicatechin (EC) and catechin (C). Consistent with our study, more catechin (C) was generated. The key enzymes ANR and LAR are directly involved in the synthesis of epigallocatechin (EGC), epicatechin (EC), and catechin (C). Previous studies have shown that after introducing LAR from *Zea mays* L into *Malus domestica* Borkh, LAR expression significantly correlates with catechins accumulation [4]. Salicylic acid could substantially increase the catechin content through induced hyperexpression of ANR in grapes [5].

Next, we detected the transcriptional profiles of genes involved in the catechin biosynthetic pathway (Fig. 1i). The qRT-PCR analysis indicated that significantly upregulated genes include *Nt4CL*, *NtCHS*, *NtF3’H*, *NtDFR*, *NtANS*, and *NtANR*. This is consistent with the accumulation of catechins, and the catechin levels in tobacco are likely increased by regulating the expression of these genes. The increase in catechin content strongly correlates with gene transcription, translation, and protein expression levels. In our research, the transgenic tobacco flowers exhibited a deeper pink color (Fig. 1g). Anthocyanin synthesis can be directly regulated by CHS, CHI, DFR, ANS, UGT, and other key enzymes. ANS is the last key enzyme in anthocyanin synthesis [9]. Compared with wild-type tobacco, *NtCHS*, *NtDFR,* and *NtANS* were significantly expressed in transgenic tobacco hosting the co-expression of *CsANS*, *CsLAR*, and *CsANR*. This may be related to the insertion of the *CsANS* gene in tobacco and the accumulation of anthocyanins in transgenic tobacco, and the high expression of this early gene indicated that the substrates and precursors are abundant for catechin and anthocyanin biosynthesis [10]. In addition, anthocyanin levels can be regulated through further metabolism or competition on parallel pathways utilizing the same substrates. Our results indicate that the co-expression of *CsANS, CsLAR*, and *CsANR* can affect the accumulation of catechins and anthocyanins and the difference in expression levels of partially synthesized genes between two transgenic lines, which may result from a synergistic effect of flavonoid pathway genes. The specific complex regulatory mechanism between them still needs further study.

To sum up, our study confirmed that the heterologous tandem co-expression of *CsANS*, *CsLAR*, and *CsANR* genes in tobacco clearly promotes catechin biosynthesis. This work provided a new perspective for understanding the synthesis and regulation mechanism of catechin in tea plant, as well as the accumulation of catechin.

## Data Availability

The authors confirm that the data supporting the findings of this study are available within the article.
